# Tracking Second Thoughts: Continuous and Discrete Revision Processes during Visual Lexical Decision

**DOI:** 10.1371/journal.pone.0116193

**Published:** 2015-02-20

**Authors:** Laura Barca, Giovanni Pezzulo

**Affiliations:** Institute of Cognitive Sciences and Technologies, National Research Council, Rome, Italy; University of California, Merced, UNITED STATES

## Abstract

We studied the dynamics of lexical decisions by asking participants to categorize lexical and nonlexical stimuli and recording their mouse movements toward response buttons during the choice. In a previous report we revealed greater trajectory curvature and attraction to competitors for Low Frequency words and Pseudowords. This analysis did not clarify whether the trajectory curvature in the two conditions was due to a continuous dynamic competition between the response alternatives or if a discrete revision process (a "change of mind") took place during the choice from an initially selected response to the opposite one. To disentangle these two possibilities, here we analyse the velocity and acceleration profiles of mouse movements during the choice. Pseudowords' peak movement velocity occurred with 100ms delay with respect to words and Letters Strings. Acceleration profile for High and Low Frequency words and Letters Strings exhibited a butterfly plot with one acceleration peak at 400ms and one deceleration peak at 650ms. Differently, Pseudowords' acceleration profile had double positive peaks (at 400 and 600ms) followed by movement deceleration, in correspondence with changes in the decision from lexical to nonlexical response buttons. These results speak to different online processes during the categorization of Low Frequency words and Pseudowords, with a continuous competition process for the former and a discrete revision process for the latter.

## Introduction

Lexical decisions have been extensively studied in cognitive psychology and neuroscience, but most studies focused on the analysis of response time under different conditions (e.g., lexical vs. non-lexical stimuli) (see [1,2]). Recent studies using continuous kinematic measures (i.e., measuring eye or mouse movements during the choice) permitted to shed light on the dynamic properties of the moment-to-moment decision process and have been applied to a number of paradigms that include numerical and colour comparisons, categorization of ambiguous figures, and semantic categorization, among others [3–7].

Continuous kinematic measures have been used to study lexical tasks, too. [8] were among the first to use this technique and showed that in an auditory lexical task mouse trajectories are attracted by phonological competitors (despite phonology was irrelevant to the task). By tracking three-dimensionally continuous motor responses, [9] revealed a complex pattern of behaviour during lexical decision, with interactive effect of word frequency and stimulus quality occurring throughout the course of a continuous motor response.

**Fig 1:**
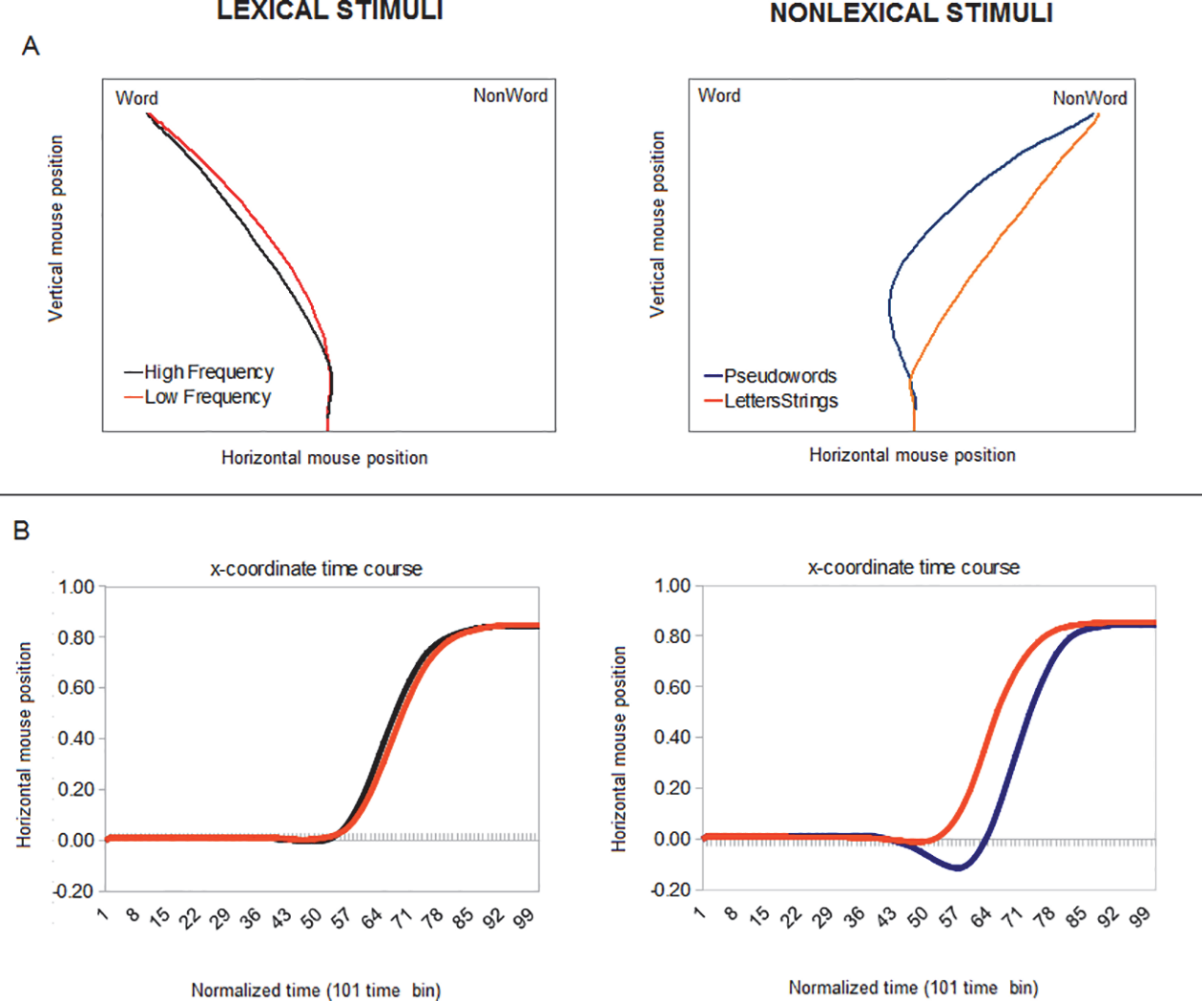
Movement trajectories for correct categorization. Panel A: mean trajectories for experimental conditions (modified by [10]). Panel B: x-coordinate time course on normalized time.

In [10] we measured participants’ kinematics (i.e., the streaming of x,y coordinates of the computer mouse) during a lexical decision task consisting in classifying written stimuli as either "lexical" or "non lexical". The study involved four kinds of stimuli varying on lexicality dimension (i.e., High Frequency words, Low Frequency words, Pseudowords, and Letters Strings). Participants performed the decision task by moving the mouse to indicate their response. Using the MouseTracker apparatus [11] we tracked continuous hand movement responses to observe the graded effects of competing items attracting the trajectory of the mouse also during trials in which the categorization was correctly executed. Mouse trajectories in categorizing Pseudowords presented large curvatures toward the alternative competing (lexical) response (see Fig. 1, panel A). The time-course of the mouse position along the horizontal plane is represented by a smooth sigmoidal curve that, in the case of Pseudowords' categorization, dip negatively toward the incorrect response option before correctly leading to the non lexical response target (Fig. 1, panel B). Trajectories for Low Frequency words presented smaller and less sharp curvatures than Pseudowords, suggesting graded competition. No such effect was present for High Frequency words and Letters Strings; see [10] for detailed analyses.

Studying lexical tasks using continuous kinematic measures permitted to extend our knowledge and revealed that several variables (e.g., phonology, word frequency) are elicited during the choice and influence it as revealed by the "curved" mouse trajectories. However, these studies leave open the issue of how exactly these factors influence the choice, and ultimately what is the nature of the lexical decision process. Even beyond the lexical decision domain, a considerable debate exists on what continuous kinematic measures reveal about the dynamical process underlying choice and what exactly produces the patterns (e.g., the mouse trajectory curvatures) observed experimentally, with alternative theories that point to a continuous competitive process among perceptual alternatives that is guided by attractor dynamics [8,12] or to a discrete perception process that feeds into a continuous motor control system [13] and can produce changes of mind [14]. According to the former (continuous competition) perspective, mouse trajectory curvatures are the fingerprints of a competitive process between the response options that continues during action performance—where essentially also an unselected response can attract the mouse movements. In this perspective, the mouse trajectory is continuously influenced by the likelihood of the alternatives, lying between the two buttons when the choices are more uncertain until eventually the competition is settled and the response button is reached. According to the latter (discrete revision) perspective, choices are always directed towards one of the alternatives and mouse trajectory curvatures result from discrete changes of mind rather than a continuous competitive process.

To disentangle these two possibilities in the case of lexical decisions, here we perform new analyses of the lexical choice experiment reported in [10] focussing on the velocity and acceleration profiles of mouse movements (note that the original study only included analyses of response time and spatial trajectories). Specifically, we ask if the curved trajectories found in both Low Frequency words and Pseudowords conditions can be characterized in terms of continuous or discrete processing. The two conditions might have different processing demands, with Pseudowords requiring the integration of ambiguous and conflicting sources of evidence (due to the resemblance of pseudowords to real words). For this reason, we hypothesize that the kinematic parameters (velocity and acceleration) might be different for Pseudowords' response compared to the other conditions (including Low Frequency words), possibly revealing different decision ‘modes’ or ‘regimes’.

## Methods

### Ethics statement

The procedure was approved by the Institute of Cognitive Sciences and Technologies of the National Research Council, ISTC-CNR of Rome. Informed consent was obtained from all participants.

### Participants

Twenty-two right-handed, neurologically healthy native Italian speakers with normal hearing and vision provided written informed consent.

### Materials and stimuli

Stimuli were 48 lexical and 48 nonlexical items, for a total of 96 trials. The lexical items were singular Italian nouns taken from the [15] database (24 High Frequency words, 24 Low Frequency words). Phonologically plausible Pseudowords (24 stimuli) were created by changing two or more letters of real low-frequency words (not included in the experimental list). Letters strings were created by randomly assembling the letters of the Italian alphabet.

**Procedure.** Participants performed a visual lexical decision task categorizing stimuli as lexical or nonlexical. They provided their response by moving the computer mouse on the selected response. During participants’ responses, the x and y coordinates of the mouse trajectories were recorded using MouseTracker. To begin each trial, participants clicked on the /START/ button located at the bottom-center of the PC screen. Then a fixation cross appeared centrally and after 300 ms was replaced by an experimental stimulus (high- or low-frequency word, pseudoword, or letters strings), which last 500 ms on the screen. Participants had to respond within 2000 ms, otherwise a /TIME OUT/ message appeared. Stimuli were presented in ARIAL font, upper case, black print on a white background. We counterbalanced across two blocks whether 'lexical' response button appear on the left and 'nonlexical' on the right, or vice versa (this was also counterbalanced across participants). See [10] for further details on the experimental procedure. The screen resolution was 1920 x 1080 pixels. The mouse was a Dell Optical USB Scroll Mouse (model N889), and the cursor location was sampled at approximately 70Hz by MouseTracker. Standard Windows mouse-sensitivity settings were used with a 1000 DPI mouse.

## Results

A total of 9% of responses were discarded from subsequent analysis (i.e., responses exceeding the time deadline accounted for 5.6% of total data, incorrect responses accounted for 3.2% of total data). Lexicality significantly affect response accuracy, with reduced accuracy for Pseudowords (mean = 1.29, standard deviation = 1.63) and Low Frequency words (mean = .33, standard deviation = .57) with respect to High Frequency words (mean = .05, standard deviation = .22) and Letters Strings (mean = .02, standard deviation = .15).

Kinematic parameters were assessed for each condition along the x-axis. At each sample the x-velocity and acceleration were calculated, these values indicate the velocity and acceleration of the mouse along the left-right dimension. ‘Movement time’ was defined as the time elapsed between stimulus onset and end of the movement. We analysed latency and amplitude of mouse movement acceleration/deceleration peaks. The ‘onset of the movement’ was determined as the first value of a sequence of at least eleven increasing points on the basis of the mouse velocity profile along the x-axes. ‘Movement velocity peak’ was determined as the maximal value in the velocity profile. Similarly, mouse ‘acceleration and deceleration peaks’ were measured as the maximal and minimal values respectively in the acceleration profile. Peak latencies were defined as the time elapsed between movement onset and maximum peak amplitude (see Table 1).

**Table 1:** Movement, acceleration and deceleration peaks.

	Velocity peak		Acceleration peak		Deceleration peak	
Stimuli	Time (ms)	Mean amplitude (pixel/sec)	Time (ms)	Mean amplitude (pixel/sec)	Time (ms)	Mean amplitude (pixel/sec)
**High Frequency**	551	.255	400–450	.0744	650–700	-.0613
**Low Frequency**	551	.25	400–450	.0569	650–700	-.0433
**Pseudowords (1** ^**st**^ **peak)**	651	.234	400–450	.0437	450–500	.0098
**Pseudowords (2** ^**nd**^ **peak)**	-	-	600–651	.0481	950–1000	-.0349
**Letters Strings**	551	.287	400–450	.0859	650–700	-.08

**Fig 2:**
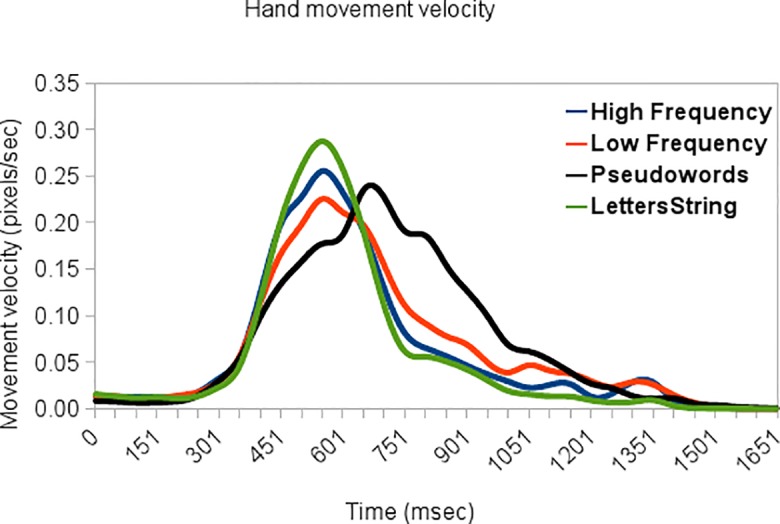
X-velocity profile for each stimulus condition.

Fig. 2 shows mean x-velocity values averaged across trajectories. X-velocity is shown here as a function of stimulus type. Inspection of the figure shows that the peak of movement response for High and Low Frequency words and for Letters Strings occurs in the same time-frame, approximately between 300 to 750 ms post-stimulus. For these three classes of stimuli, velocity peak is quite sharp and its amplitude is modulated by their lexicality, with increasing amplitude from Low Frequency words to High Frequency words and Letters Strings. This result is in line with previously reported results on overall response time. The time-frame of occurrence of Pseudowords' movement peak is much wider, rising approximately at the same time as the other stimuli (around 300ms post-stimulus) and lasting until 1000ms post-stimulus. Longer latency of the movement suggests that during the processing of Pseudowords some processing is tacking place that interferes with (or at least affects) the execution of the movement and the underlying decision.

Cross-correlation analysis has been used to test similarities between time-series of different conditions (we have used the programming language R, http://www.r-project.org/). The cross-correlation function is the correlation between the times-series shifted against one another as a function of number of observations of the offset. The range of cross-correlation value is -1 to 1 such that the closer the value is to 1, the more closely the information sets are, and values of 0 indicating no correlation. A total of 50 time-lags over 1950ms were used, meaning that each lag corresponds to a shift in approximately 40ms. With a time-lag of zero (that is no shifting between time-series), values of cross-correlation were high when correlating the time-series of High Frequency words with Low Frequency words (.94) and Letters String (.96), and correlating Low Frequency words with Letters Strings (.9). The time-series of Pseudowords have a lagged correlation with the time-series of the other categories, so that the highest positive values with High Frequency words (.89) occurs at time-lag -2, with Low Frequency words (.93) at time-lag -1, and with Letters String (.87) at time-lag -2, meaning that to be positively coupled with the other categories the time-series of Pseudowords have to be anticipated of approximately 80ms in the case of High Frequency words and Letters Strings, and approximately 40ms in the case of Low Frequency words.

To assess differences between conditions we used Bayesian estimation, a probabilistic approach allowing quantification of parameter estimates and uncertainty in the form of the posterior distribution. Markov chain Monte-Carlo (MCMC) sampling methods were used to accurately approximate the posterior distributions of the estimated parameters (the approach and its advantages over classical t-test are fully explained in [16]). For computing the Bayesian inference we have used the programming language R (http://www.r-project.org/) and Markov chain Monte Carlo (MCMC) sampling language called JAGS [16]. Results are reported in Table 2.

**Table 2:** Bayesian Parameter Estimation on velocity data.

Two-sample comparison	muDiff	ProbDiffmu>0	sigmaDiff	ProbDiffsigma>0	effSz
**High Frequency-Low Frequency**	-.004	24%	-.006	19%	-.21
**High Frequency-Pseudowords**	-.001	49%	-.01	11%	-.04
**Pseudowords-Letters Strings**	.005	79%	.01	96%	.23
**High Frequency-Letters Strings**	.005	88%	.003	76%	.34
**Low Frequency-Letters Strings**	.01	95%	.01	94%	.49
**Low Frequency-Pseudowords**	.002	62%	-.004	28%	.07

Summaries of posterior distributions for the derived parameters: difference in means (muDiff), difference in standard deviation (sigmaDiff) and effect size (effSz). Probabilities that the difference in means (probDiffmu>0) and standard deviation (probDiffsigma>0) are greater than zero are also reported.

Results of Bayesian estimation showed greater certainty in estimating the difference in mean velocity when comparing posterior distributions for High Frequency words and Letters String (the mean difference is greater than zero by a probability of 88%), Low Frequency words and Letters String (95% of credible values are greater than zero), and comparing Pseudowords with Letters String (79% of credible values are greater than zero). Similar results were observed considering differences in standard deviation of posterior distributions. Table 2 also reports the mode of the distribution of the effect sizes (the effect size is computed for each credible combination of means and standard deviations). For High Frequency words and Letters String distribution of credible effect sizes has a mode of .34, a mode of .49 for Low Frequency words vs. Letters String, and .23 comparing Pseudowords with Letters String. As for the other contrast, differences in means and standard deviation of posterior distributions are smaller and the model reveals great uncertainty in estimating the differences.

The significance of the differences between amplitudes of peak velocity response was further determined using one factor Analysis of Variance on selected time windows. Two time windows were built in order to capture the observed peaks (tw1: 400–700ms post stimulus; tw2 600–900 ms post stimulus). TukeyHSD test for multiple comparison of means have been used to check differences among different levels of the variable. ANOVA on tw1 with Stimulus Category as factor, showed lexicality modulation of velocity (F (1, 3) = 4.92, MSE = .009; p<.005), with higher amplitude of Letters Strings' velocity peak than Low Frequency (p adjusted<.05, difference = .008) and Pseudowords (p adjusted <.005, difference = .01). No other comparison were significant (all ps<.1) As for the second time window, the analysis showed main effect of stimulus type (F (1, 3) = 96.8, MSE = .207; p<.001). No difference emerged between High Frequency words and Letters Strings, whereas all other difference were significant (p adjusted <.005): High Frequency words produce higher amplitude than both Low Frequency words (difference = -.10) and Pseudowords (difference = -.05), and Pseudowords have significantly higher amplitude than Low frequency (difference = .04) words and Letters Strings (difference = .05).

**Fig 3:**
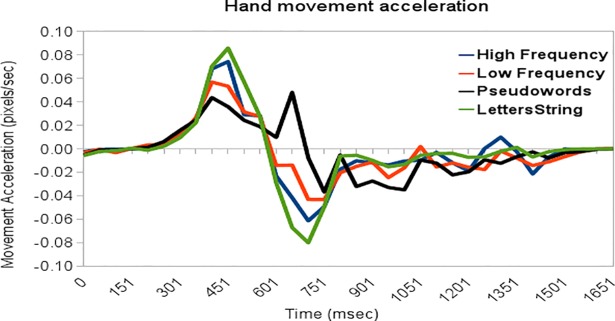
X-acceleration profile for each stimulus condition.

Fig. 3 shows the movement acceleration profile for each condition. Graphical profile of movement kinematics revealed that movements acceleration peaks for High/Low Frequency words and Letters String exhibit the same butterfly profile, with movement peak acceleration occurring within similar time window (400–450 ms post stimulus onset) as deceleration peak (650–700 ms post stimulus onset).

Different from the other categories, Pseudowords exhibit a different profile with a double acceleration peak. The first acceleration peak occurring within the same time frame as the other categories, with a deflection of the movement not crossing the zero line but rising up again between 600–650 ms post stimulus onset. Movement deceleration of this second peak occurred 950–1000 ms post stimulus onset.

Cross-correlation between conditions time-series have been conducted. With a time-lag of zero, values of cross-correlation were high when correlating the time-series of High Frequency words with Low Frequency words (.94) and Letters String (.96), and correlating Low Frequency words with Letters Strings (.9). The time-series of Pseudowords have a lagged correlation with the time-series of the other categories, so that the highest positive values occurs with Low Frequency words (.73) at time-lag 0, and at time-lag -2 with High Frequency words (.65). The highest positive correlation with Letters Strings occurs at time-lag -1, which is small (.28), suggesting moderate correlation between time-series. Results indicate that to be positively coupled with the other categories the time-series of Pseudowords have to be anticipated of approximately 80ms in the case of High Frequency. Table 3 reports the Bayesian estimation of the differences between conditions.

**Table 3:** Bayesian Parameter Estimation on acceleration data.

Two-sample comparison	muDiff	ProbDiffmu>0	sigmaDiff	ProbDiffsigma>0	effSz
**High Frequency-Low Frequency**	.002	72%	1.15e-05	51%	.16
**High Frequency-Pseudowords**	2.5e-05	51%	-.0007	44%	.01
**Pseudowords-Letters Strings**	-.0006	40%	.003	92%	-.07
**High Frequency-Letters Strings**	-1.65e-05	51%	.002	87%	-.01
**Low Frequency-Letters Strings**	-.002	20%	.003	92%	-.23
**Low Frequency-Pseudowords**	-.002	33%	-.0007	41%	-.13

Summaries of posterior distributions for the derived parameters: difference in means (muDiff), difference in standard deviation (sigma Diff) and effect size (effSz). Probabilities that the difference in means (probDiffmu>0) and standard deviation (probDiffsigma>0) are greater than zero are also reported.

Overall differences in means are very small and the estimates are not reliable. Bayesian estimates shows greater credibility for differences in standard deviation of posterior distributions, especially contrasting Low Frequency words and Pseudowords with Letters String (92% of values exceeding zero difference between standard deviations, in both cases), and High Frequency words with Letters Strings (87% of values exceeding zero difference between standard deviations) though the parameter estimate is less precise.

Values of x-acceleration were entered into the same One-Way Anova described above for x-velocity values. Two time periods were selected: the first time period (tw1) starts at 300ms until 600ms post stimulus presentation, and capture the first event of the movements; the second time period (tw2) starts at 600 ms and ends at 800ms post stimulus and capture the second event of the movements. One-way ANOVA (with Stimulus Category as factor) on acceleration data of tw1 did not showed effects of stimulus category (F (1, 3) = 1.25, MSE = .029; p>.1). Differences emerged on tw2 data (F (1, 3) = 12.9, MSE = .004; p<.001), with higher amplitude of Pseudowords's acceleration with respect to the other categories (i.e., High Frequency words: p adjusted <.001, difference = -.005; Low Frequency words: p adjusted = .06, difference = -.003; Letters Strings: p adjusted <.001, difference = -.006), and between Letters Strings and Low Frequency words (p adjusted <.005, difference = .004). No other differences were significant (all ps>.1).

### Pseudowords' trajectories reversal

**Fig 4:**
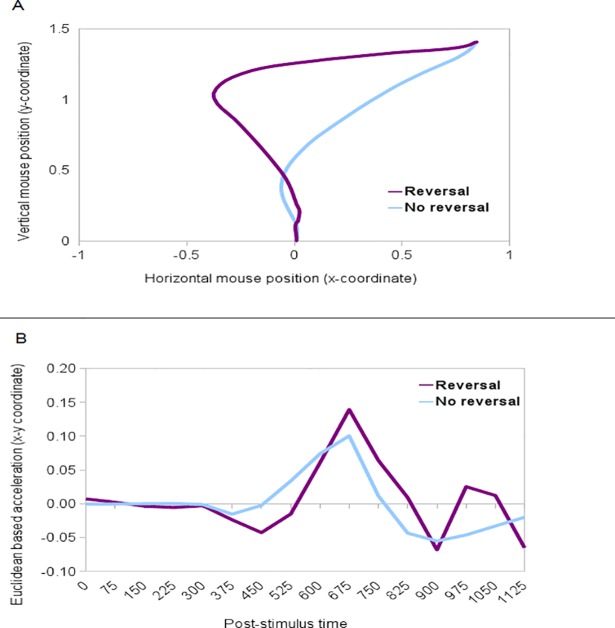
Pseudowords’ reversal. Mean trajectories (panel A) and Euclidean based x-y acceleration profile (panel B) for trials marked as having a reversal or no reversal.

To examine the possibility that different response populations might account for the double peak observed in pseudowords' acceleration profile, additional analysis have been performed focusing on pseudowords' trajectories presenting reversal or abrupt shifts. Abrupt shifts were identified using a quantitative method devised in [3], with trajectories exceeding an MD threshold of 0.9 coded as “reversal” trajectories and those underneath such threshold coded as “no reversal”. Reversal (n 88) and no reversal (n 331) trajectories and their Euclidean-based acceleration profile (from both x-y coordinates) are plotted in Fig. 4.

The mean trajectories of “no reversal” group (Fig. 4, panel A) showed continuous attraction toward the lexical response. Differently, the mean trajectories for “reversal” group showed strong attraction toward the lexical response with a sharp mid-flight correction leading to the correct categorization.

The acceleration profile (Fig. 4, panel B) of the two groups appeared different. “Reversal” and “no reversal” trajectories first exhibit a deflection in the movement followed by positive spike around 700ms. For “reversal” trajectories only, a second positive peak follows the deceleration phase with lower amplitude than the first one. Bayesian parameter estimation (Table 3) was used for group comparison.

Bayesian estimates show great credibility for differences in means contrasting spatial information for “no reversal” and “reversal” trajectories. The 95% Highest Density Interval of the difference of means falls well above zero, and 95% of the credible values are greater than zero. Therefore is it possible to conclude that the groups’ means are credibly different. Standard deviations of the two groups are not credibly different (with only 24% of credible values being greater than zero).

As for the acceleration data, differences in means and standard deviation of the groups are less credible (see Table 4). Different time windows were built in order to capture the events observed in “reversal” and “no reversal” acceleration profile (tw1: 300–525ms; tw2: 526–750ms; tw3: 751–975ms; tw4: 976–1275ms). T tests were used to identify periods in the acceleration profiles where the two groups differ significantly (p < .05; two-tailed t test). Significant differences emerged only in tw3 (t.test = 2.2, df = 108.9, p<.05) and tw4 (t.test = -2.2, df = 112.7, p<.05). Thus, the acceleration profiles were significantly different between “reversal” and “no reversal” trajectories between 750 and 1275 ms (p<.05), where the second peak of acceleration occurs for “reversal” trajectories.

**Table 4:** Bayesian Parameter Estimation on reversal and no reversal Pseudoword's trajectories.

Two-sample comparison NoReversal-Reversal	muDiff	ProbDiffmu>0	sigmaDiff	ProbDiffsigma>0	effSz
**X values**	.089	95%	.027	25%	.23
**Acceleration values**	-.006	37%	-.008	26%	-.07

Summaries of posterior distributions for the derived parameters: difference in means (muDiff), difference in standard deviation (sigmaDiff) and effect size (effSz). Probabilities that the difference in means (probDiffmu>0) and standard deviation (probDiffsigma>0) are greater than zero are also reported.

Reversal trajectories presented also a higher fluctuation on the horizontal axis (x-flips mean: 0.94, standard deviation: 0.48) than no reversal trajectories (x-flips mean: 0.39, standard deviation: 0.54), indicating more uncertainty in the decision.

**Fig 5:**
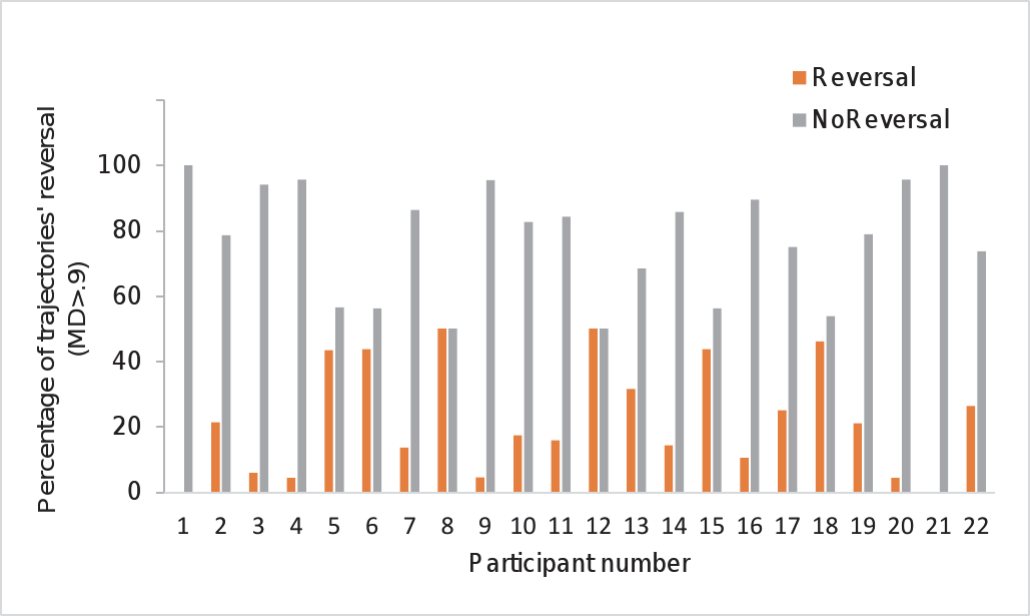
Reversal and no reversal Pseudowords' trajectories for individual participants.

The difference in reversal and no reversal trajectories suggests that a mixture of different strategies might be used to categorize Pseudowords, thus we also examined individual participants to check for within- or between subjects’ differences (see Fig. 5).

Among the group of participants, only two of them did not have any reversal trajectory (subject 1 and 21), and five participants have a small percentage of reversal (less than 10% of pseudowords' responses). Nine participants have 14% to 32% of reversal, and the remaining six participants have similar percentage of reversal and no reversal.

The pattern of subject variability provide additional evidence for the occurrence of different processes taking place in categorizing legal pseudowords: graded-competition vs “changes of mind” and online revision processes.

## Discussion

We conducted a kinematic study of mouse movements during lexical decisions (i.e., classifying a stimulus as a word or non-word) with the aim to understand how such decisions unfold dynamically in time and whether the underlying decision process can be described as a continuous competition between response alternatives or a discrete process with sharp changes of mind [12–14].

We have previously reported the analysis of the spatial aspects (i.e., trajectories) of the mouse movements, which revealed spatial attraction toward the opposite category for Low Frequency words and Pseudowords, which have higher ambiguity compared to High Frequency words and Letters Strings [10]. Here we focus instead on velocity and acceleration profiles of the responses, showing that these are significantly modulated by lexicality.

To provide a converging line of evidence for differences in timings between our four types of stimuli, we ran several types of analyses in which we computed the mean cross-correlation function between time-series of the stimuli, Bayesian estimation of differences between posterior distributions, and Analysis of Variance of selected time windows located around the peaks in velocity and acceleration profiles. The analysis of the velocity profile during the choice reveals that the different stimulus categories have different peaks. All except Pseudowords peak at the same time, but with different amplitude. This plausibly indicates a different level of uncertainty associated with the different stimuli, mirroring the reaction time and mouse curvature results reported in [10]. Pseudowords reach peak velocity significantly later in time, reflecting the overall difficulty of the decision. Over the 1950ms window for which cross-correlations were computed, Pseudowords' hand movement velocity (and acceleration) time-series shows a phase lag of 40–80 ms compared to the other stimuli.

The analysis of hand acceleration profiles reveals a single peak of acceleration occurring 300ms post stimulus presentation for High Frequency words, Low Frequency words, and Letters Strings. Differently, trajectories in the Pseudowords conditions can be divided into two classes: “reversal” (where we observe abrupt shifts in the direction of the trajectory) and “no reversal” (where no abrupt shifts are observed). In the case of “reversal” Pseudowords trajectories (only), hand acceleration and deceleration parameters displayed a double peak. This pattern of results is not observed in the case of “no reversal” Pseudowords trajectories. Although a double peak of acceleration does not necessarily imply a “change of mind”, our results suggest that at least in certain cases participants classifying Pseudowords revised their decision abruptly from the initial decision to classify stimuli as lexical to the successive (correct) non-lexical choice. Our method for extracting trial-by-trial response strategies also revealed great subject variability in the participants’ responses to Pseudowords, with different subjects using “reversal” and “no reversal” trajectories in different proportions, suggesting that they might employ different strategies to deal with task uncertainty. These results have important implications for computational models of decision-making and lexical processing, which we discuss below.

Perceptual decision-making tasks (including lexical decision) are often interpreted in terms of dynamic models of decision-making, in which evidence is continuously accumulated for the competing alternatives (lexical vs. non-lexical). There are many variants of "diffusion-to-bound" models, which include drift-diffusion [17], attractor models [18], and neural "race" models [19,20]. This family of models is supported by single-unit neuronal data in perceptual decision tasks [21]. A related but distinct family of models emphasizes dynamic competition in the context of Bayesian inference schemes, which include bottom-up accumulation of evidence and top-down expectation-based dynamics [22,23]. Although the diffusion-to-bound framework provides a coherent process model of decision and a good fit of response time, it tends to separate decision and action processes because a default assumption is that action starts when decisions are completed (i.e., after passing a decision bound or threshold). However, several perceptual decision-making studies using continuous kinematic measures of performance have shown that actions (e.g., the mouse movements for clicking one of two buttons) are initiated before decision is completed and revised along the way [5,7,24]. To explain the on-line dynamics of choice, another family of models has been developed in which decision and action are not segregated processes but rather there is a continuous flow of activation from the former to the latter [25]. An even more radical view is that decision and action are conducted in parallel and can influence one another [26–30]. One such models that has been used to explain lexical tasks describes decision as a continuous and graded competition between the response alternatives, which act as "attractors" in attractor networks [29]; the competition takes place during action performance producing the characteristic curved trajectories observed in uncertain situations. However, this view is not unchallenged. An alternative view is that decisions are discrete processes and the curved trajectories depend on the fact that they fed into a continuous motor execution process [13]. Sharp revisions of an initial revision or "changes of mind" can be seen as extensions of the standard diffusion-to-bound model [14]. Here there is no graded competition between the alternatives but instead an initial commitment to one response that can be followed by a sharp deviation towards the other response alternative (a "change of mind") on the basis of evidence that was not initially considered.

The results of our experiment speak directly to this debate, suggesting that different decision modes might be expressed within the same experiment—and even the same experimental condition (Pseudowords)—depending on stimuli characteristics and task uncertainty. The comparison of data on Low Frequency words and Pseudowords in our experiment illustrates the presence of both graded competition (in both conditions) and changes of mind (in the latter condition). Indeed, both Low Frequency words and Pseudowords show significant mouse curvatures (see Fig. 1) but at variance from the other stimuli some trajectories in the Pseudowords condition have two acceleration peaks (see Fig. 3), strongly suggesting a true "change of mind" during the decision and the associated need to decelerate and accelerate again to change movement direction to correctly report the choice. It is possible to speculate that the late velocity peak for Pseudowords (see Fig. 2) is due to the fact that the initial trajectory corresponds to a very high uncertain decision, and only in correspondence with the revised choice the uncertainty has the same level as the other conditions.

## Conclusions

We report two distinct online choice processes (or regimes) during lexical decisions: a continuous competition process for Low Frequency words and a discrete revision process for Pseudowords. These results speak to leading models of dynamic decision-making in the literature, which tend to see these regimes as mutually exclusive alternatives [8,12–14]. It remains to be established what underlying (neural and computational) process might produce the two regimes that we observe and under which circumstances we should expect the former or the latter. It has been proposed that, at least in principle, dynamic attractor models might produce both continuous and discrete choices under different uncertainty conditions [12], but whether this applies to our task remains to be tested more directly in future research. It is also worth noting that none of these models includes realistic movement dynamics such as the fact that biomechanic constraints prevent an instantaneous trajectory revision (which implies among the other things that changes of mind can produce two peaks of acceleration). When biomechanical constraints are considered, it becomes apparent that slower reaction time could be due to various factors, which include the time needed to accumulate evidence up to a threshold and resolve the uncertainty, and the time needed for changing hand trajectory, which can include decelerations and accelerations. The former but not the latter factors are considered in existing diffusion-to-bound models of decision-making. Models of decision-making that include movement dynamics have been recently developed but are not widely applied so far [6,26,28,31]; some of these models make novel predictions such as the fact that the costs of reaching a target or changing mind should be considered as an integral aspect of the decision-making process, which implies that action planning and performance become part and parcel of the choice rather than outputs [26,27,32,33,34]. In parallel with the development of experimental techniques that nowadays permit to track the on-line dynamics of choice we need to design increasingly more realistic models that incorporate action dynamics, biomechanic constraints, and other factors that matter in ecologically valid choice contexts.

Our results speak also specifically to psycholinguistic studies and models, which have so far neglected or at least rarely investigated the on-line dynamics of lexical decisions [1,2]. By using a continuous measure of performance [4–7,29] we have mapped choice uncertainty into different parameters of the movement (curvature, velocity and acceleration profile) and not only on reaction time as usually done. This method permitted us to investigate the micro-dynamics of lexical and non-lexical stimuli processing, revealing important differences in their processing regimes that are masked if one only considers a mono-dimensional measure such as reaction time.

An open question is what are the factors that enter in the lexical choice and produce the different pattern of results for Pseudowords compared to the other conditions. As we discussed earlier, current decision-making models assume that the competitive process is guided by the continuous accumulation of evidence [17]. Psycholinguistic models suggest that during a lexical decision such evidence is collected via a stochastic processing consisting in "matching" stimulus features with stored memory representations. In the REM model [35] these representations include trace vectors of feature values; in the ‘interactive account’ [36] the representations are maintained in the reciprocal connections of cortical hierarchies. These models do not fully specify what is the nature of these features, but these could include for example visual, orthographic, phonological, and semantic information. In principle, the decision might consider all (and other) sources of information. However, they might be available at different moments in time because semantic information requires more time to be retrieved [10]. In keeping, the evidence accumulation process for Low Frequency words and Pseudowords might be essentially different. In Low Frequency words, the initially available (e.g., visual, orthographic, or phonological) information points towards the "lexical" alternative, although with some uncertainty; the successively available (e.g. semantic) information points to the same alternative and thus does not call for a revision process. Rather, in Pseudowords the initially available information points towards the "lexical" alternative due to the resemblance with real words but the successively available (e.g. semantic) information points to the "non-lexical" alternative with high certainty, thus requiring a drastic revision process. This hypothesis points to a non-stationary evidence accumulation process that, once modelled computationally, has been shown to produce revision dynamics in the decision-to-bound process [19]; in our set-up, these revision processes might be manifest in the overt mouse movements rather than only in the decision process as normally assumed. Developing computational models that combine non-stationary evidence accumulation and realistic action dynamics will permit testing this hypothesis.
